# Clinical Interest in Exome-Based Analysis of Somatic Mutational Signatures for Non-Small Cell Lung Cancer

**DOI:** 10.3390/cancers16173115

**Published:** 2024-09-09

**Authors:** Morgane Peroz, Hugo Mananet, Nicolas Roussot, Courèche Guillaume Kaderbhai, Valentin Derangère, Caroline Truntzer, François Ghiringhelli

**Affiliations:** 1 Platform of Transfer in Biological Oncology, Georges-Francois Leclerc Cancer Center—UNICANCER, 21000 Dijon, France; mperoz@cgfl.fr (M.P.); hmananet@cgfl.fr (H.M.); vderangere@cgfl.fr (V.D.); 2 Unité de Formation et de Recherche des Sciences de Santé, University of Burgundy-Franche-Comté, 21000 Dijon, France; 3 Unité Mixte de Recherche de l’Institut National de la Santé Et de la Recherche Médicale (INSERM) 1231, 21000 Dijon, France; 4 Department of Medical Oncology, Georges François Leclerc Cancer Center—UNICANCER, 21000 Dijon, France; nroussot@cgfl.fr (N.R.); cgkaderbhai@cgfl.fr (C.G.K.)

**Keywords:** non-small cell lung cancer, whole exome sequencing, somatic mutational signatures, immune checkpoint inhibitors, prognostic biomarkers

## Abstract

**Simple Summary:**

Non-small cell lung cancer (NSCLC) remains the leading cause of cancer-related mortality. This study investigates the clinical interest of whole exome sequencing for analyzing somatic mutational signatures in patients with advanced or metastatic NSCLC treated with the current standard of care. Investigating somatic mutational signatures as well as structural variations, we evaluated the association between genomic features and patient outcomes in a cohort of 132 patients. This study identified specific signatures associated with poor response to immune checkpoint inhibitor (ICI) therapy and chemotherapy, potentially aiding treatment selection and identifying patients unlikely to benefit from these approaches.

**Abstract:**

Background: Non-small cell lung cancer (NSCLC) remains the leading cause of cancer-related mortality. This study investigates the clinical interest of whole exome sequencing (WES) for analyzing somatic mutational signatures in patients with advanced or metastatic NSCLC treated with the current standard of care. Methods: Exome sequencing data and clinical characteristics from 132 patients with advanced or metastatic NSCLC were analyzed. Somatic mutational signatures including single base substitutions (SBSs), double base substitutions (DBSs), and copy number signatures were evaluated. Structural variations including tumor mutational burden (TMB), the number of neoantigens, TCR clonality, homologous recombination deficiency (HRD), copy number alterations (CNAs), and microsatellite instability (MSI) score were determined. The association between these genomic features, NSCLC subtypes, and patient outcomes (progression-free and overall survival) was evaluated. Conclusions: Exome sequencing offers valuable insights into somatic mutational signatures in NSCLC. This study identified specific signatures associated with a poor response to immune checkpoint inhibitor (ICI) therapy and chemotherapy, potentially aiding treatment selection and identifying patients unlikely to benefit from these approaches.

## 1. Introduction

Non-small cell lung cancer (NSCLC) remains the leading cause of cancer-related mortality worldwide [1]. Despite significant advances in treatment modalities, challenges persist in accurately predicting patient outcomes and tailoring therapies. In this context, understanding the underlying mutational landscape of NSCLC has emerged as a critical area of research.

Somatic mutation assessment is essential in the management of NSCLC [2,3]. At diagnosis, the presence of oncogenic addiction notably with EGFR mutation but also with ROS, ALK, RET, or NTRK fusion is essential to determine prognosis and to guide the first line of treatment. In patients without oncogenic addition, with the exception of RAS mutant tumors, first-line metastatic disease treatment is based on the use of immune checkpoint inhibitor (ICI) therapy, either alone or in combination with chemotherapies. The therapeutic decision is based on programmed death-ligand 1 (PD-L1) status. However, by its capacity to determine tumor mutation burden (TMB), next-generation sequencing (NGS) could also be used to improve the prediction of immunotherapy efficacy.

The implementation of large NGS panel testing in recent years has led to the rise of mutational signature analysis, a powerful tool that decodes the patterns of mutations within cancer genomes. These signatures, characterized by specific types and frequencies of mutations, provide valuable insights into the mutagenic processes responsible for tumorigenesis. Using data from more than 23,000 cancer patients, the International Cancer Genome Consortium (ICGC)/The Cancer Genome Atlas (TCGA) Pan-Cancer Analysis of Whole Genomes (PCAWG) Consortium [4] has revealed many mutational signatures across the spectrum of human cancer types. They proposed a consensual classification and developed SigProfiler, a compilation of publicly available bioinformatics tools addressing all the steps needed for signature identification. Four mutational signatures based on DNA sequencing were considered in this study, resulting in single base substitutions (SBSs), double base substitutions (DBSs), small insertions and deletions (IDs), and copy number alterations (CNAs).

In addition, genomic instability scores could be determined like microsatellite instability (MSI), homologous recombination deficiency (HRD), and copy number alteration (CNA) scores.

However, the relation between these variations and NSCLC subtypes and/or prognosis is not fully elucidated. This study explores the rationale for analyzing these parameters using whole exome sequencing performed during the management of advanced or metastatic NSCLC.

## 2. Materials and Methods

### 2.1. Study Population

Patients with locally advanced unresectable or metastatic solid cancer treated with ICIs at the Georges-François Leclerc Cancer Center (Dijon, France) and who had exome sequencing were included in this retrospective single-center study. All patients were prospectively included in the EXOMA1 and EXOMA2 trials (respectively, NCT02840604 and NCT04614480). The exome sequencing was performed prospectively according to each EXOMA trial protocol.

Genomic analyses were performed at the Georges-Francois Leclerc Cancer Center in the Genomic and Immunotherapy Medical Institute, Dijon, France. All patients provided written informed consent for the trial and genomic analysis. After informed consent, patients had a consultation with a genetic counselor before the constitutional exome analysis.

The dedicated analysis for the purposes of the present study was performed retrospectively and was not the main purpose of the original EXOMA trials.

Patient and tumor characteristics were collected, namely sex, age, WHO performance status (PS), smoking history, histologic type, sites of metastasis, medical treatments, and best response to first-line treatment. The best response assessment was based on computed tomography (CT) scans using the RECIST 1.1 criteria. For details of the study design, see Supplementary Figure S1.

The database was registered with the French National Commission on Informatics and Liberty (CNIL). This study was conducted in accordance with French legislation and the Declaration of Helsinki, with approval from the relevant institutional review boards.

### 2.2. Sample Selection

Whole exome sequencing (WES) analysis is performed in routine care in our center in order to identify potentially targetable mutations for second-line therapy. Before patients consented to WES of their tumoral tissue, they were informed by their oncologist. Physicians selected an archival tumor sample (primary or metastasis) for genomic analysis. At the physician’s discretion, a new tumor biopsy could be proposed to the patient. Germline testing was performed after counseling by a clinical geneticist.

### 2.3. Sample Analysis

DNA was isolated from archival tumor tissue using the Maxwell 16 FFPE Plus LEV DNA Purification kit (Promega, Madison, WI, USA). DNA from whole blood (germline DNA) was isolated using the Maxwell 16 Blood DNA Purification kit (Promega) according to the manufacturer’s instructions. The quantity of extracted genomic DNA was assessed by a fluorimetric method with a Qubit device.

### 2.4. Whole Exome Capture and Sequencing

A total of 200 ng of genomic DNA was used for library preparation, using the Agilent SureSelectXT reagent kit (Agilent Technologies, Santa Clara, CA, USA). The totality of the enriched library was used in the hybridization and captured with the SureSelect All Exon v5 or v6 (Agilent Technologies) baits. Following hybridization, the captured libraries were purified according to the manufacturer’s recommendations and amplified by polymerase chain reaction (12 cycles). Normalized libraries were pooled, and DNA was sequenced on an Illumina NextSeq500 device using 2 × 111 bp paired-end reads and multiplexed. Tumor and germline DNA sequencing generated mean target coverages of 78× and 90×, respectively, and a mean of more than 90% of the target sequence was covered with a read depth of at least 10× for somatic DNA.

### 2.5. Exome Analysis Pipeline

Reads in the FASTQ format were aligned to the reference human genome GRCh37 using the Burrows–Wheeler aligner (BWA v0.7.17). Local realignment was performed using the Genome Analysis Toolkit (GATK v4.1.3.0). Duplicate reads were removed using Picard v.2.5. Single-nucleotide variants (SNVs) were detected using a validated pipeline that integrates mutation calls from three different mutation callers. SNVs were called with VarScan (v2.4.3) [5] and Mutect (v1.1.7) [6], and insertion/deletions (indels) were called with VarScan and Strelka (v2.9.2) [7].

TMB was calculated using the number of significant SNVs (with Untranslated Transcribed Regions, synonyms, introns, and intergenic SNVs filtered out) divided by the number of megabases covered at a defined level. To identify tumor-specific mutant peptides, pVAC-Seq (personalized Variant Antigens by Cancer Sequencing) [8] was used (pVACtools v1.5.4). This computational workflow compares and differentiates the epitopes found in normal cells against the neoepitopes specifically present in tumor cells to predict neoantigens. pVAC-Seq is based on HLA typing obtained by HLAminer (v1.4) [9]. The microsatellite instability (MSI) score was computed using MSIsensor (v0.5) [10]. Copy number alterations (CNAs) were inferred using the Titan algorithm [11]. CNA score was computed as described by Danlos et al. [12]. The homologous recombination deficiency (HRD) score was obtained through the scarHRD [13] pipeline. The presence and quantitation of T-cell receptor (TCR) clones were determined using the MixCR software (v3.0.4) [14], available at http://mixcr.milaboratory.com/ (accessed on 1 March 2023). SigProfilerAssignment [15] was used to perform mutational signature analysis; specifically, single base substitutions (SBSs), double base substitutions (DBSs), small insertions and deletions (IDs), and copy number alteration (CNA) signatures were determined.

### 2.6. Statistical Analysis

Patient characteristics are described as median and interquartile range (IQR) for continuous variables and as number and percentage (%) for qualitative variables.

Characteristics were compared using the Chi-squared test or Fisher’s exact test for qualitative variables, or the Wilcoxon test for continuous variables, as appropriate. *p*-values were adjusted using Benjamini–Hochberg [16] False Discovery Rate (FDR) correction, and adjusted *p*-values < 0.05 were considered statistically significant.

Progression-free survival (PFS) was calculated as the time from the start of immunotherapy until disease progression and was censored at two years. Overall survival (OS) was defined as the time from the start of immunotherapy until death from any cause.

Survival analysis was performed using the survival R library. The prognostic value of the different variables was tested using univariate and multivariate Cox models for PFS and OS. Survival probabilities were estimated using the Kaplan–Meier method, and survival curves were compared using the log-rank test.

Variables with unadjusted *p*-values < 0.10 by univariate analysis were selected for multivariate analysis. For signature variables, a composite score was then estimated based on the corresponding linear predictor of the multivariate Cox model. These scores were then dichotomized (High vs. Low) based on the cut-off value determined by the median.

Data were analyzed using R (version 4.0.3) statistical software (http://www.R-project.org/, accessed on 1 March 2023), and graphs were prepared with Prism 9 (GraphPad, San Diego, CA, USA).

## 3. Results

### 3.1. Patient Characteristics

We analyzed exome and clinical data from 132 patients prospectively included in the EXOMA 1 and 2 trials between 2015 and 2020 and treated for advanced or metastatic NSCLC. Blood and tumor samples were available for all of the patients.

The detailed clinical characteristics of the patients are described in Table 1 for the overall study cohort and in Supplementary Table S1 for the EXOMA 1 and 2 cohorts.

Among the 132 patients analyzed, 16 (12%) patients had squamous NSCLC, and 115 patients had non-squamous NSCLC; NSCLC type was missing for one patient. No patient with squamous NSCLC had oncogenic addition with related therapeutic indication. In patients with non-squamous NSCLC, 10 had EGFR mutant tumors, 23 had no G12C KRAS mutated tumors, and 15 had G12C KRAS mutated tumors, whereas 67 patients were considered as WT, i.e., non-KRAS and non-EGFR mutated tumors.

The PD-L1 tumor proportion score (TPS) was available for 112 patients, and the status was ≥50% in 43 (38%) patients.

**Figure 1:**
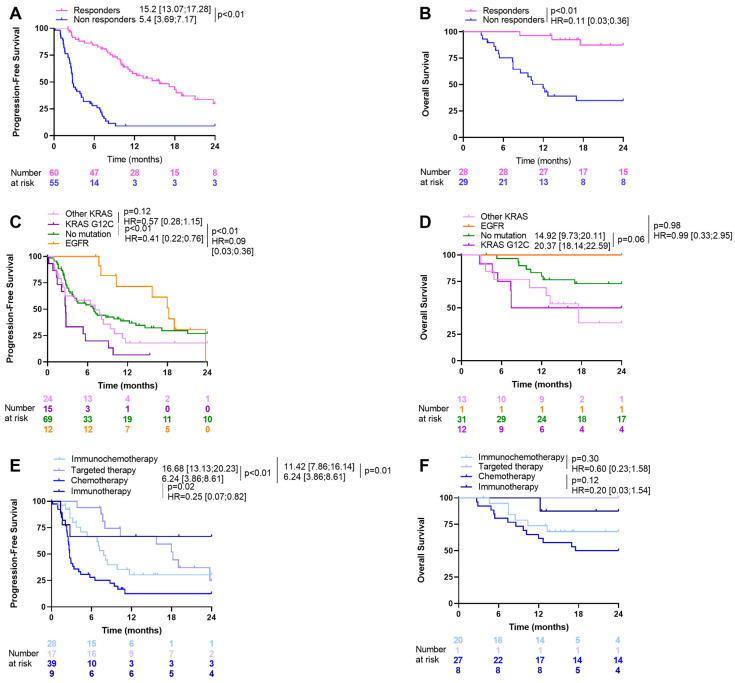
Kaplan–Meier curves for progression-free and overall survival according to response status (**A**,**B**), actionable mutation pattern (**C**,**D**), and type of treatment (**E**,**F**).

In the overall population, 9 patients were treated in the first line by immunotherapy alone, 40 patients by chemotherapy alone, 28 patients by chemoimmunotherapy, and 17 patients by targeted therapies (12 were treated with osimertinib, 1 with alectinib, and 3 with brigatinib). The RECIST criteria were available for 116 patients. Among these, 60 (52%) were considered responders (complete or partial response), and 56 (48%) experienced stable or progressive disease (non-responders) after the first-line regimen. Median overall survival in the population was 34.8 [17.6, 58.2] months, and progression-free survival was 7.1 [4.5, 9.9] months. There was a strong relationship between the response to first-line treatment choice outcomes in terms of PFS and OS (Figure 1A,B).

Regarding PFS and OS, we observed a strong prognostic difference when patients were analyzed according to their actionable mutation pattern, i.e., EGFR mutant tumors, WT EGFR/RAS tumors, KRAS G12C mutant tumors, and other KRAS mutant tumors (Figure 1C,D). In contrast, we observed no difference in prognosis between squamous and non-squamous NSCLC tumors. Similarly, treatment choice was associated with prognosis, with the best outcome observed among patients treated with target therapies and the worst prognosis in patients treated with chemotherapy alone (Figure 1E,F).

### 3.2. Analysis of Genomic Scores

Using exome analysis, we generated various scores, e.g., MSI, HRD, CNA scores, TMB, neoantigen number, and TCR clonality for each patient. The genomics characteristics are described in Table 2 for the overall study cohort and in Supplementary Table S2 for the EXOMA 1 and 2 cohorts.

TMB and the number of neoantigens were correlated. Similarly, CNA and HRD scores were strongly correlated (Figure S2).

**Figure 2:**
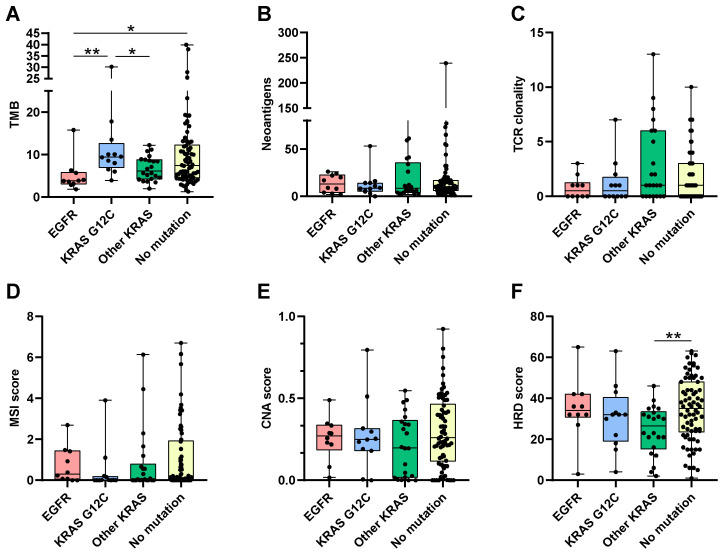
Boxplots representing the distribution of tumor mutational burden (**A**), the number of neoantigens (**B**), TCR clones (**C**), MSI (**D**), CNA (**E**), and HRD (**F**) scores, according to actionable mutation pattern. *: Wilcoxon *p*-value < 0.05; **: Wilcoxon *p*-value < 0.01.

Boxplots show the distribution of these metrics in function of the cancer type and mutations (Figure 2). TMB was lower in EGFR mutant tumors. KRAS G12C tumors had higher TMB than other KRAS mutant tumors (Figure 2A).

The number of neoantigens did not differ between cancer-type groups. TCR clonality was also reduced in EGFR mutant tumors in comparison to other tumor types (Figure 2C).

MSI and CNA scores were very low for all patients with no statistically significant differences between subtypes (Figure 2D,E). HRD score was lower in patients with non-KRAS G12C tumors than in the other groups (Figure 2F). None of these parameters were associated with either PFS or OS.

### 3.3. Analysis of Mutational Signatures

**Figure 3:**
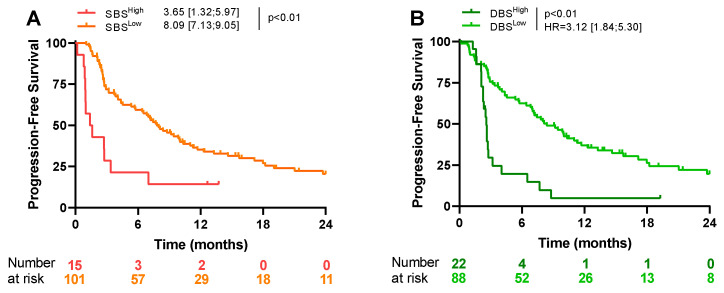
Kaplan–Meier curves for progression-free survival, according to SBS-associated signatures (**A**), and according to DBS-associated signatures (**B**).

Using SBS signatures, 96 different patterns of mutation were previously isolated. After FDR correction, we observed that SBS5 was significantly associated with objective responses to first-line treatments (Figure S3). This signature is notably related to tobacco smoking. When looking at first-line PFS, only a high presence of SBS7a, SBS19, SBS24, SBS28, and SBS89 signatures was associated with outcome by univariate analysis. Combining these markers identified a population with a very poor prognosis (Table 3 and Figure 3A).

When looking at the distribution of this prognostic signature between treatment groups or molecular subtypes, we observed that EGFR mutant tumors were all in the group with good prognosis, while the group with poor prognosis included more patients treated with immunotherapy as monotherapy (Table 4 and Table 5).

Using DBS signatures, 78 different signatures were generated after the concurrent modification of two consecutive nucleotide bases. By multivariate analysis, DBS6, DBS9, and DBS15 were associated with poor PFS. Combining these markers isolated a population with a very poor prognosis (Table 3 and Figure 3B).

When looking at the distribution of this prognostic signature between treatment groups or molecular subtypes, we observed that the groups with poor prognosis included more patients treated with chemotherapy alone (Table 4 and Table 5).

Lastly, none of the 48 CN signatures were related to the outcome.

## 4. Discussion

This study explored the potential of exome sequencing for analyzing somatic mutational signatures in patients with advanced or metastatic NSCLC treated with the current standard of care. By identifying distinct mutational signatures associated with treatment response and prognosis, our findings offer valuable insights for personalized medicine approaches in NSCLC management.

With the rapid development of NGS panels and the decreasing cost of genomic sequencing, exome or genome testing is currently developing broadly in multiple types of cancer. However, the current ESMO recommendations for molecular testing in NSCLC only recommend analysis of a dedicated panel of genes related to currently approved target therapies (EGFR, ROS1, ALK, KRAS, BRAF, RET, MET, HER2, and NTRK). For immunotherapy only, TMB determination and KEAP1 and STK11 mutation analysis are proposed as an option [17,18]. The ESCAT (ESMO Scale for Clinical Actionability of molecular Targets) recommendations are then used to classify variations [19]. Previous data have suggested a modest interest in large panel or exome sequencing to improve the management of metastatic NSCLC [20,21,22]. Indeed, large panels highlighted ESCAT class II, III, and IV mutations, which, for the most part, were not targetable. However, large panels were slightly less efficient for class I ESCAT mutations thus suggesting that it is only a complementary tool to classical panel sequencing.

In addition to mutation annotations, several metrics can be generated from exome data. First, the tumor mutational burden and the presence of neoantigens are classical biomarkers of response to immunotherapy in NSCLC [23]. In this study, as expected, we found an association between TMB and KRAS mutational status as previously reported [24,25,26]. We did not findany association between TMB or neoantigen number and outcome. HRD, CNA score, and MSI score were very low in all patients of the cohort, reflecting the carcinogenesis mode of NSCLC, which rarely presents homologous or mismatch repair deficiency [27].

Somatic mutations are generated by the activities of endogenous and exogenous mutational processes, with each process exhibiting a characteristic mutational pattern, termed the mutational signature [4,28,29]. Prior studies have demonstrated that mutations are not uniformly distributed across the genome and that most mutational signatures are affected by the topographical features of the human genome [30,31]. Previous data have also underlined that in some contexts, a mutational signature can be associated with the prognosis or response to treatment [32]. For example, in the context of NSCLC, a smoking signature is associated with a better response to immunotherapy.

Using a global assessment of SBSs and DBSs, we observed that SBS mutational signatures were linked with poor prognosis in patients receiving immunotherapy while DBSs could predict poor response to chemotherapy. These signatures could be used to identify patients unlikely to respond to such therapies, allowing for earlier exploration of alternative treatment options and potentially improving patient outcomes.

While these findings are promising, some limitations need to be acknowledged. The study’s retrospective nature and relatively small sample size imply that the findings warrant validation in larger, prospective cohorts. Additionally, the functional mechanisms underlying the associations between specific mutational signatures and treatment response or prognosis require further investigation.

## 5. Conclusions

This study demonstrates the potential of exome sequencing for analyzing somatic mutational signatures in NSCLC. The identification of signatures associated with treatment response and prognosis paves the way for personalized medicine approaches in NSCLC management. Further validation and mechanistic studies are essential to establish the clinical utility of mutational signature analysis in guiding treatment decisions and improving patient outcomes.

## Figures and Tables

**Table 1:** Clinical characteristics in the overall study cohort (*N* = 132).

Variables	Whole Cohort *N* = 132
Age	64 (58, 72)
Unknown	23
Sex	
Male	62 (57%)
Female	47 (43%)
Unknown	23
Smoking status	
Never smoker	15 (15%)
Current smoker	35 (34%)
Former smoker	52 (51%)
Unknown	30
WHO performance status	
0	16 (15%)
1	62 (58%)
2	24 (23%)
3	4 (3.8%)
Unknown	26
Brain metastasis	
0	79 (72%)
1	31 (28%)
Unknown	22
Lymph node metastasis	
0	22 (20%)
1	88 (80%)
Unknown	22
Lung metastasis	
0	86 (78%)
1	24 (22%)
Unknown	22
Bone metastasis	
0	58 (53%)
1	52 (47%)
Unknown	22
PDL1 (cutoff at 50%)	
<50%	69 (62%)
≥50%	43 (38%)
Unknown	20
Histological type	
Adenocarcinoma	95 (73%)
Carcinoma	16 (12%)
Other	20 (15%)
Unknown	1
Histological type	
Squamous	16 (12%)
Non-squamous	115 (88%)
Unknown	1
Treatments	
Chemotherapy	40 (43%)
Chemoimmunotherapy	28 (30%)
Immunotherapy	9 (9.6%)
Targeted therapy	17 (18%)
Unknown	38
Response	
Complete response	9 (7%)
Partial response	51 (44%)
Stable disease	26 (22%)
Progressive disease	30 (26%)
Unknown	16

*N* (%): Median (IQR), Fisher’s exact test. PDL1: Programmed Death-Ligand 1.

**Table 2:** Genomic characteristics in the overall study cohort (*N* = 132).

Variables	Whole Cohort *N* = 132
TMB score	6.9 (4.3, 10.8)
Unknown	14
Number of neoantigens/Mb	9 (6, 17)
Unknown	17
Number of strong neoantigens/Mb	1 (0, 3)
Unknown	17
TCR clonality	1 (0, 2)
KRAS/EGFR mutation	
KRAS G12C	15 (11%)
Other KRAS	24 (18%)
EGFR	12 (9.1%)
No mutation	81 (61%)
HRD score	32 (22, 42)
Unknown	13
MSI score	0.08 (0.00, 1.15)
Unknown	14
CNA score	0.25 (0.09, 0.40)
Unknown	13

TMB: tumor mutational burden; HRD: homologous recombination deficiency; MSI: microsatellite instability; CNAs: copy number alterations. *N* (%): Median (IQR); TCR: T-cell receptor; KRAS: Kirsten rats arcomaviral oncogene homolog; EGFR: Epidermal Growth Factor Receptor.

**Table 3:** Univariate and multivariate Cox models for progression-free survival and SBS/DBS signatures.

Variables	Univariate			Multivariate		
	HR	95%CI	*p*-Value	HR	95%CI	*p*-Value
SBS7a	1.10	[1.04; 1.16]	0.002	1.08	[1.01; 1.14]	0.02
SBS19	1.06	[1.01; 1.11]	0.013	1.07	[1.02; 1.12]	0.005
SBS24	1.01	[1.00; 1.01]	0.008	1.00	[0.99; 1.01]	>0.9
SBS28	1.07	[1.03; 1.11]	<0.001	1.08	[1.04; 1.12]	<0.001
SBS89	1.01	[1.00; 1.01]	<0.001	1.01	[1.00; 1.01]	<0.001
DBS6	1.24	[1.05; 1.47]	0.011	1.28	[1.08; 1.51]	0.004
DBS9	1.75	[1.17; 2.62]	0.006	1.87	[1.25; 2.81]	0.002
DBS15	1.22	[1.02; 1.46]	0.032	1.25	[1.05; 1.50]	0.014

HR: hazard ratio; CI: confidence interval; SBS: single base substitution, DBS: double base substitution.

**Table 4:** The number of patients receiving each treatment type in the two survival groups created according to SBS and DBS signatures.

	Treatment	Chemotherapy	Chemoimmunotherapy	Immunotherapy	Targeted Therapy
Group					
SBS^Low^		32 (32%)	24 (24%)	5 (5%)	17 (17%)
SBS^High^		5 (33%)	4 (27%)	4 (27%)	0 (0%)
DBS^Low^		21 (24%)	26 (30%)	6 (7%)	13 (15%)
DBS^High^		13 (60%)	2 (9%)	2 (9%)	2 (9%)

SBS: single base substitution; DBS: double base substitution.

**Table 5:** Repartition of molecular subtypes in the two survival groups created according to SBS and DBS signatures.

	Mutation	KRAS G12C	Other KRAS	EGFR	No Mutation
Group					
SBS^Low^		12 (12%)	21 (21%)	12 (12%)	56 (56%)
SBS^High^		3 (20%)	2 (13%)	0 (0%)	10 (66%)
DBS^Low^		11 (12%)	18 (20%)	9 (10%)	50 (57%)
DBS^High^		3 (14%)	5 (23%)	2 (9%)	12 (55%)

SBS: single base substitution; DBS: double base substitution; KRAS: Kirsten rats arcomaviral oncogene homolog; EGFR: Epidermal Growth Factor Receptor.

## Data Availability

Data are available from authors upon reasonable request.

## Supplementary Materials

The following supporting information can be downloaded at https://www.mdpi.com/article/10.3390/cancers16173115/s1, Figure S1: Flowchart of study design; Figure S2: Correlation matrix of six genomic variables; Figure S3: Boxplot representing distribution of SBS5 signature, according to response to treatment; Supplementary Table S1: Patient clinical characteristics in EXOMA1, EXOMA2, and whole cohorts; Supplementary Table S2: Patient genomic characteristics in EXOMA1, EXOMA2, and whole cohorts.
