# Supplementary material for: Clinical Interest in Exome-Based Analysis of Somatic Mutational Signatures for Non-Small Cell Lung Cancer
Source: Cancers (Basel). 2024 Sep 9;16(17):3115. doi: 10.3390/cancers16173115 (PMC11393922; doi:10.3390/cancers16173115)
Supplement: Supplementary file 1 [file cancers-16-03115-s001.zip › New Microsoft Word Document.pdf]

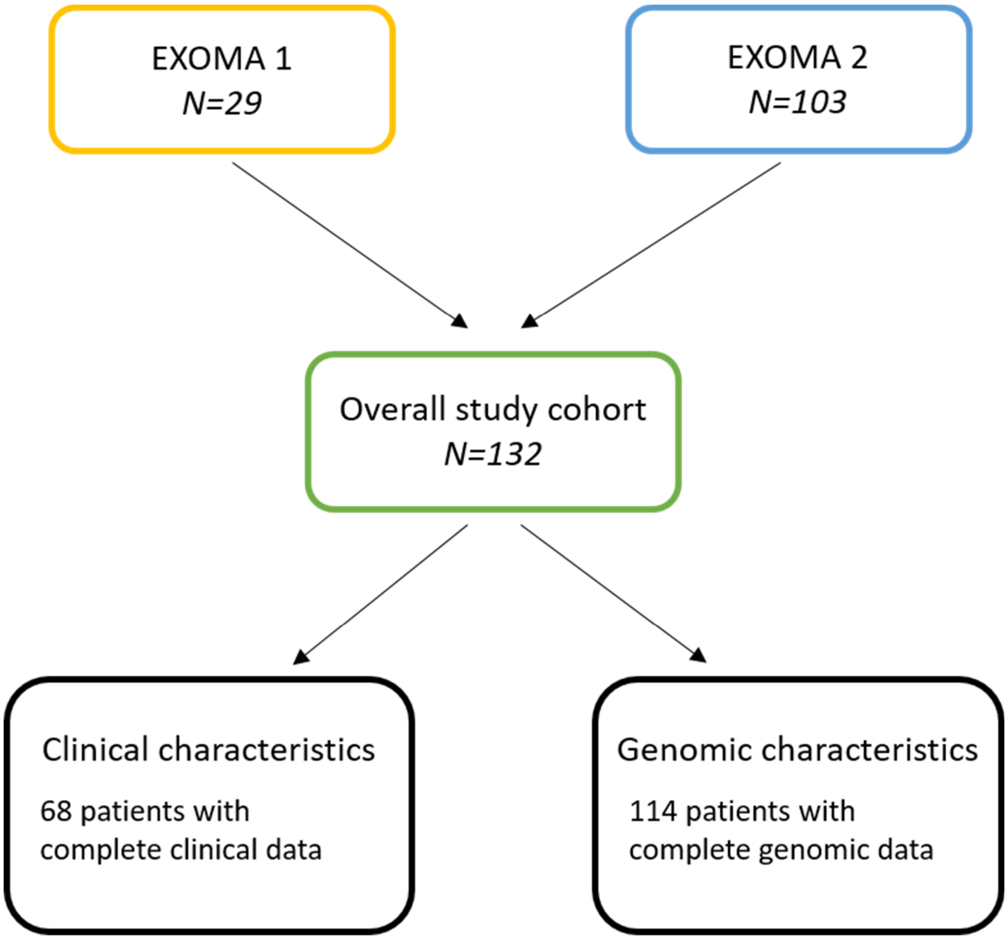

Figure S1. Flowchart of study design.

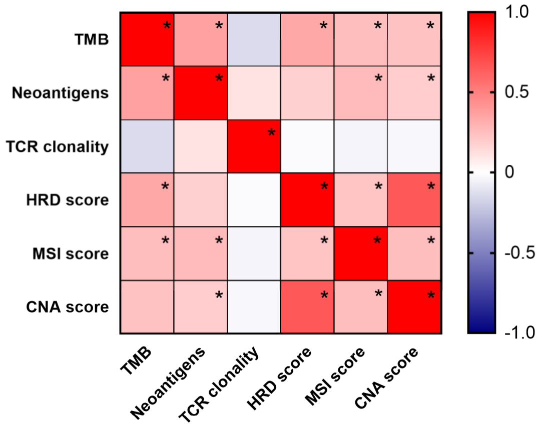

Figure S2: Correlation matrix of six genomic variables.

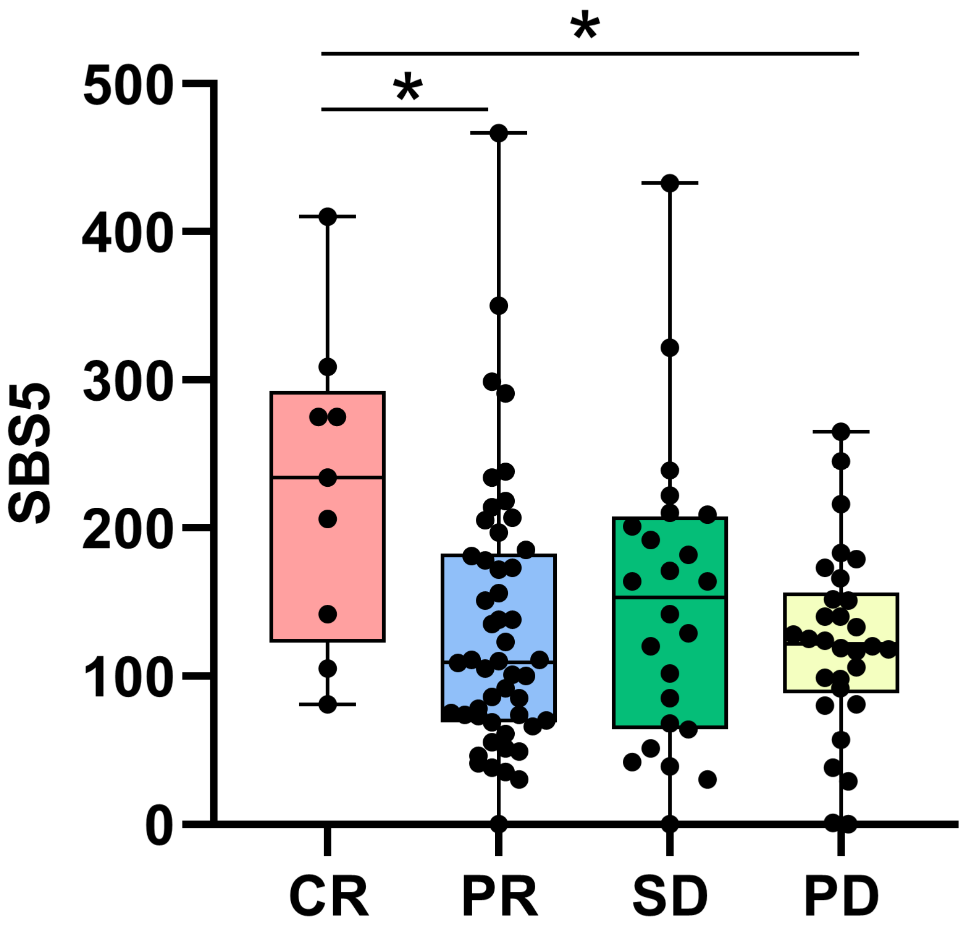

Figure S3: Boxplot representing distribution of SBS5 signature, according to response to treatment.
