# Supplementary material for: Clinical Interest in Exome-Based Analysis of Somatic Mutational Signatures for Non-Small Cell Lung Cancer
Source: Cancers (Basel). 2024 Sep 9;16(17):3115. doi: 10.3390/cancers16173115 (PMC11393922; doi:10.3390/cancers16173115)
Supplement: Supplementary file 1 [file cancers-16-03115-s001.zip › SupplementaryTable1.pdf]

Variables		Whole cohort *N*=132	EXOMA1 *N*=29	EXOMA2 *N*=103	p-Value	Ajusted p-Value
Age		64 (58, 72)	61 (58, 69)	66 (60,72)	0.2	0.4
	Unknown	23	0	23		
Sex					0.8	0.9
	1	62 (57%)	17 (59%)	45 (56%)		
	2	47 (43%)	12 (41%)	35 (44%)		
	Unknown	23	0	23		
Smoking status					0.7	0.8
	0	15 (15%)	2 (8.3%)	13 (17%)		
	1	35 (34%)	9 (38%)	26 (33%)		
	2	52 (51%)	13 (54%)	39 (50%)		
	Unknown	30	5	25		
WHO performance status					>0.9	>0.9
	0	16 (15%)	4 (14%)	12 (15%)		
	1	62 (58%)	16 (57%)	46 (59%)		
	2	24 (23%)	7 (25%)	17 (22%)		
	3	2 (1.9%)	0 (0%)	2 (2.6%)		
	4	2 (1.9%)	1 (3.6%)	1 (1.3%)		
	Unknown	26	1	25		
Cerebral metastasis					0.6	0.7
	0	79 (72%)	22 (76%)	57 (70%)		
	1	31 (28%)	7 (24%)	24 (30%)		
	Unknown	22	0	22		
Lymphnod metastasis					0.8	0.9
	0	22 (20%)	5 (17%)	17 (21%)		
	1	88 (80%)	24 (83%)	64 (79%)		
	Unknown	22	0	22		
Lung metastasis					0.7	0.8
	0	86 (78%)	22 (76%)	64 (79%)		
	1	24 (22%)	7 (24%)	17 (21%)		
	Unknown	22	0	22		
Bone metastasis					0.2	0.4
	0	58 (53%)	18 (62%)	40 (49%)		
	1	52 (47%)	11 (38%)	41 (51%)		
	Unknown	22	0	22		
PDL1 (cutoff at 50%)					<0.001	<0.001
	0	69 (62%)	5 (20%)	64 (74%)		
	1	43 (38%)	20 (80%)	23 (26%)		
	Unknown	20	4	16		

Histological type					
Adenocarcinoma	95 (73%)	23 (79%)	72 (71%)		
Carcinoma	16 (12%)	5 (17%)	11 (11%)	0.008	0.2
Other	20 (15%)	1 (3.4%)	19 (19%)		
Unknown	1	0	1		
Histological type					
Squamous	16 (12%)	5 (17%)	11 (11%)	0.3	0.4
Non-squamous	115 (88%)	24 (83%)	91 (89%)		
Unknown	1	0	1		
Treatments					
Chemotherapy	40 (43%)	23 (79%)	17 (26%)		
Immuno-chemotherapy	28 (30%)	1 (3.4%)	27 (42%)	<0.001	<0.001
Immunotherapy	9 (9.6%)	4 (14%)	5 (7.7%)		
Targeted therapy	17 (18%)	1 (3.4%)	16 (25%)		
Unknown	38	0	38		
Response					
Complete response	9 (7%)	3 (10%)	6 (6.9%)		
Partial response	51 (44%)	6 (21%)	45 (52%)	0.001	0.006
Stable disease	26 (22%)	5 (17%)	21 (24%)		
Progressive disease	30 (26%)	15 (52%)	15 (17%)		
Unknown	16	0	16		

N (%) : Median (IQR), Fisher's exact test ; Wilcoxon rank sum test; * *p*-values were adjusted using Benjamini–Hochberg FDR correction; FDR: false discovery rate.

Supplementary Table S1 : Patient clinical characteristics in EXOMA1, EXOMA2 and whole cohort.
