# Supplementary material for: Clinical Interest in Exome-Based Analysis of Somatic Mutational Signatures for Non-Small Cell Lung Cancer
Source: Cancers (Basel). 2024 Sep 9;16(17):3115. doi: 10.3390/cancers16173115 (PMC11393922; doi:10.3390/cancers16173115)
Supplement: Supplementary file 1 [file cancers-16-03115-s001.zip › SupplementaryTable2.pdf]

Variables	Whole cohort<br>*N*=132	EXOMA1<br>*N*=29	EXOMA2<br>*N*=103	p-Value	Ajusted<br>p-Value
TMB score	6.9 (4.3 ; 10.8)	7.1 (5.7, 11.0)	6.7 (4.1, 10.8)	0.2	0.3
Unknown	14	1	13		
Number of neoantigens	9 (6, 17)	8 (6, 12)	10 (6, 20)	0.3	0.4
Unknown	17	1	16		
Number of strong neoantigens	1 (0, 3)	1 (0, 2)	1 (0, 4)	>0.9	>0.9
Unknown	17	1	16		
TCR clonality	1 (0, 2)	0 (0, 1)	1 (0, 3)	0.007	0.017
KRAS/EGFR mutation				0.11	0.2
KRAS G12C	15 (11%)	7 (24%)	8 (7.8%)		
Other KRAS	24 (18%)	5 (17%)	19 (18%)		
EGFR	12 (9.1%)	1 (3.4%)	11 (11%)		
No mutation	81 (61%)	16 (55%)	65 (63%)		
HRD score	32 (22, 42)	32 (16, 43)	32 (23, 42)	0.5	0.5
Unknown	13	0	13		
MSI score	0.08 (0.00, 1.15)	0.03 (0.00, 0.08)	0.25 (0.01, 1.97)	0.005	0.017
Unknown	14	1	13		
CNA score	0.25 (0.09, 0.40)	0.25 (0.03, 0.36)	0.25 (0.10, 0.41)	0.5	0.5
Unknown	13	0	13		

TMB : Tumor Mutational Burden ; HRD : Homologous recombination deficiency ; MSI : Microsatellite instability ; CNA : copy number alterations. *N* (%) : Median (IQR), Fisher's exact test ; Wilcoxon rank sum test; * *p*-values were adjusted using Benjamini–Hochberg FDR correction; FDR: false discovery rate.

Supplementary Table S2. Patient genomic characteristics in EXOMA1, EXOMA2 and whole cohort.
